# A Word about Hospitals

**Published:** 1870-01

**Authors:** 


### Editorial.

### *A Word About Hospitals.*

WE see by the daily press that Cook County Hospital is crowded with patients, and for a long time we have known that the deficient space allotted to the sick of the city and county has been guarded by "red tape," to a degree that has rendered admission to its privileges, practically, a matter of extreme difficulty.

A man, wounded and friendless, picked up in the street by the police, stands a better chance of dying in the station house or the Armory than of finding lodging and care in the county castle of Hygeia.

No blame is herein intended for the efficient medical and surgical staff which attend at the county building.

The city and county are deeply in debt. The erection of the new and expensive wings of the Court House, the new Bridewell, Normal School building, etc., etc., tax, already, the abilities of the people, and the financial skill of the powers that be, to an extent that causes them to shrink from the attempt to provide new and capacious hospitals. Yet, this latter is an imperious necessity.

There is scarcely a city on the continent, or throughout Christendom, where there is so little provision for the sick poor. Private effort will vainly attempt to supply the want.

Chicago *must* have increased hospital accommodations. This is a fact too patent, too palpable, to need argument; and being assumed, we beg leave to submit to the profession and the community, that the want can easily and satisfactorily be filled.

For hospitals, the main demand is space — plenty of land and no more brick or stone castles. There is not, in this age of the world, a more unpardonable anachronism than the vast architectural monstrosities which every where, almost, in the cities, are piled up and labeled hospitals.

Let us have no more of them. Spend all the spare money for land — there is plenty of it on sale in the county — and then put up cheap buildings, constructed in accordance with modern ideas of hospital proprieties.

Plain wooden structures, not over two stories in height, with no cellars or foundations, save the necessary piers of support. No one roof to cover more than twenty-five, or, at the utmost, fifty beds. Long, narrow — with windows upon both sides in abundance — with the wind blowing under, and around, and over them.

We insist, the wooden barracks employed as hospital wards in the late war, were infinitely preferable to the masses of brick, stone and mortar, built for all time, labeled hospitals, but which are rather charnel houses.

Put up the buildings cheaply enough so that should they become pestilential, notwithstanding judicious care, they may be pulled down or burned up, with no one to lament the waste.

The war experience has taught civilians much in this matter, and we had thought the day of castellated hospitals had gone by. But it would seem that the authorities still think they must adhere to the deplorable uses of antiquity.

Let us have no more "Guy's" — not even any more wings or "lean-tos" to the present Cook County Hospital. Better pull that down, or sell it for a brewery, and have something in accordance with the teachings of modern science.

Let us have no more strangers, or even vagrants, dying in the Armory, Bridewell or station houses, because there is no room for the sick or wounded poor in the hospitals of this great city of Chicago.

***Rush Medical College.***

The Annual Commencement exercises occur on Wednesday evening, February 2nd, at the lower lecture room of the College.

The third Annual Reunion of the Alumni Association of Rush Medical College will occur on Commencement day, Feb. 2. The meeting will convene in the lecture-room of the College, at 10 A.M. The annual address will be given by Dr. Ames, president of the Association.

Diplomas will be conferred on an unusually large class.

***Portrait of Prof. Gross.***

We are indebted to the enterprising Editor of the Philadelphia *Reporter*, for a finely-executed engraved likeness of the veteran surgeon and teacher, Prof. S. D. Gross. Subscribers to the *Reporter* will find it in the second January number, and *artist's proofs* may be obtained, at $1.00 each, by addressing S. W. Butler, M.D., 115 South Seventh Street, Philadelphia.

***Responsibility.***

It is, perhaps, unnecessary to say to the majority of readers that the Editors do not hold themselves responsible for the opinions of correspondents. All that we require of them is, matter of professional interest, written in *good faith.*

All Book Notices, Editorials, etc., the Senior Editor assumes personal responsibility for, unless the authorship is otherwise indicated by name or initial.

Relieved entirely from the cares of publication, we commence the new year with renewed zeal and determination to maintain the Journal in the front rank of medical periodical literature.

***Ingratitude.***

Sometime since, we took occasion to compliment, in warm terms, a very prettily got up compilation (elegantly illustrated) of metters connected with the Hip-Joint and its Dislocations. But it appears, because we took the liberty to call the author's attention to a single glaring instance of failure to give appropriate credit, he has insinuated that we had not read his book.

He should recollect what Sydney Smith said — never read a book before reviewing it, as reading it is apt to *prejudice* you.

Unfortunately, we did read the book referred to, sufficiently to ascertain its real merits, and have nothing to take back or add to what we have written.

***Caution.***

ALL persons indebted to the JOURNAL, for past subscriptions or advertisements, are cautioned against paying any money to any person therefor except to the undersigned, or his order. The last volume of the JOURNAL came very near to grief, under its then publisher, and the undersigned is making every effort to repair the damages thus sustained. J. ADAMS ALLEN.

